# Diabetic retinopathy and OCT angiography: clinical findings and future perspectives

**DOI:** 10.1186/s40942-017-0062-2

**Published:** 2017-03-13

**Authors:** Jose Mauricio Botto de Barros Garcia, David Leonardo Cruvinel Isaac, Marcos Avila

**Affiliations:** Federal University of Goias, Av. Primeira Avenida, S/N, Rua 234, 38, Apto 1011, Setor Leste Universitario, Goiania, GO CEP 74605-020 Brazil

**Keywords:** Macula, Retina, Imaging, Optical coherence tomography

## Abstract

In diabetic retinopathy (DR), macular involvement can present as either macular edema or ischemia. Fluorescein angiography remains the gold standard in the evaluation of retinal vascular perfusion and diagnosis of macular ischemia. However, it is a costly, time-consuming technique, it requires venipuncture, and reports of anaphylaxis and death related to fluorescein injections have been documented, despite their rarity. Optical coherence tomography (OCT) provides a fast and non-invasive method to assess retinal structures at a microscopic level. OCT angiography permits the noninvasive study of retinal and choroid circulation via motion contrast imaging. Split-spectrum amplitude decorrelation angiography combined with OCT angiography has furthered the understanding of retinal and choroidal vascular diseases, allowing the evaluation of retinal microvasculature and identification of subsequent disorders, including DR. Previous studies using OCT angiography have demonstrated that it may demonstrate DR findings such as microaneurysms, arteriolar wall staining, retinal neovascularization, and intraretinal microvascular abnormalities. The purpose of this article is to describe and discuss different concepts regarding OCT angiography, as well as its role in the diagnosis of DR and maculopathy.

## Background

Approximately 347 million people worldwide have diabetes mellitus (DM) [1]. The worldwide prevalence of DM is predicted to grow to 430 million patients by 2030 [2]. Diabetic retinopathy (DR), a diabetic microangiopathy, is characterized by microaneurysms (MAs), capillary nonperfusion, and ischemia within the retina [3–6]. It may cause several complications, such as diabetic macular edema (DME) and diabetic macular ischemia (DMI) [1, 7–11]. In particular, capillary nonperfusion impairs the nutrition of the neuroglial tissues in the retinal parenchyma, and the resultant hypoxia increases the expression of vascular endothelial growth factor (VEGF), which promotes both angiogenic responses and vascular permeability [12]. Diabetic maculopathy is caused by a combination of both VEGF-mediated factors and inflammatory mediators [13–15].

DME occurs when high glucose levels cause capillaropathy that damages the blood supply to the retina. A hallmark of this disease is alterations in the blood-retinal barrier that is characterized by pericyte loss and endothelial cell–cell junction breakdown [16]. Numerous treatments have been established to manage DME, such as focal or grid photocoagulation and antiangiogenic therapy, which have been recently shown to yield good results in the treatment of DME [7, 11, 17].

Fluorescein angiography is broadly recognized as an important tool in the diagnosis and treatment of DR [15]. However, it requires venipuncture, and reports of anaphylaxis and death related to contrast injections, although rare, have been documented [18]. In addition, the technique is costly and time-consuming, requiring up to 10 min for framing acquisition [19–21]. Nevertheless, it remains the gold standard in the analysis of DR features.

The development of optical coherence tomography (OCT) has revolutionized ophthalmology and provides a rapid and noninvasive method to assess retinal structures at the microscopic level [20]. Spectral domain OCT (SD OCT) has become an important tool with which to manage patients with DR [17].

OCT angiography permits the noninvasive imaging of retinal and choroidal circulation via motion contrast imaging [21, 22]. This relatively novel imaging technique obtains high-resolution volumetric blood flow information and generates angiographic images in a matter of seconds [5, 6, 21, 22]. OCT angiograms are resampled with OCT B-scans from the same area, simultaneously allowing the assessment of structure and blood flow [21, 23].

The high speed of SD OCT compared with ime-domain OCT (TD OCT) has made the imaging of both the structure and blood flow possible [24, 25]. Several OCT-derived techniques have been successfully developed to image in vivo human eye microcirculation, such as phase-variance OCT (PV OCT), phase contrast OCT (PC OCT), and split-spectrum amplitude decorrelation angiography (SSADA), which will be discussed herein [24–26].

The purpose of this article is to describe and discuss different concepts regarding OCT angiography, as well as its role in the diagnosis of DR and maculopathy.

## OCT angiography: technology applied to retinal disorders

Since it was first described to capture ophthalmic images in 1991, OCT has increasingly been used in clinical practice and ophthalmic research [27]. OCT angiography is a further step in OCT technology that allows microvascular assessment by detecting blood flow. Different OCT angiography platforms and segmentation algorithms have been described, with the AngioVue software of RTVue XR Avanti (Optovue, Fremont, CA) being the first commercially available OCT angiography system [21, 22, 24]. This device obtains volumetric scans of 304 × 304 A-scans at 70,000 A-scans per second in approximately 3.0 s. The 3 × 3-mm OCT angiograms appear to provide higher resolution images than the currently available imaging techniques [20]. SSADA aims to improve the signal to noise ratio (SNR) [22, 24, 28, 29]. Its application with OCT angiography instrumentation has furthered the understanding of retinal and choroidal vascular diseases.

Using SSADA, Spaide and co-workers demonstrated in vivo a distinct superficial capillary plexus (SCP) and deep capillary plexus (DCP), the latter of which includes the intermediate plexus [28, 30]. Thus, OCT angiography could be used to enrich the understanding of ischemic conditions that may affect the different layers of retinal microcirculation, such as cotton wool spots, acute macular neuroretinopathy (AMN), paracentral acute middle maculopathy (DCP ischemia), and macular telangiectasia (MacTel) type 2 [28, 31–33]. Reports applying swept-source OCT technology (SS OCT) in patients with MacTel 2 have already shown structural abnormalities in the inner retina, such as retinal cavitation with draping of the internal limiting membrane and abnormalities in the outer retina, including disruptions of the photoreceptor ellipsoid segment that were not previously appreciated by fluorescein angiography [33].

The AngioPlex OCT angiography instrument (Carl Zeiss Meditec, Dublin, CA) improved the CIRRUS HD OCT scanning rate to 68,000 A-scans per second and introduced a tracking software known as FastTrac retinal-tracking technology. The standard scanning patterns available include 3 × 3 and 6 × 6 mm OCT angiograms. The angiographic images are generated using a complex algorithm that analyzes differences in both the intensity and phase information from repeated B-scans in the same position. This process is repeated at multiple adjacent positions to generate an *en face* flow volume. This algorithm is known as OCT microangiography-complex (OMAG) [34]. AngioPlex and AngioVue have been compared in clinical practice, and it was demonstrated that AngioPlex requires a shorter time and provides a higher number of images available for analysis with fewer motion artifacts [35]. Another SD OCT angiography device is the NIDEK RS-3000 (NIDEK, Aichi, Japan), which provides scans that range from 3 × 3 to 9 × 9 mm. Moreover, up to a 12 × 9-mm panorama image can be automatically generated.

SS OCT technology uses longer wavelength infrared light (1050 nm) than conventional SD OCT. This technology enables improved penetration into tissue and imaging through optical opacities and is invisible to the subject. The DRI OCT (Triton SS OCT; Topcon, Tokyo, Japan) imaging systems, including the Atlantis prototype technology, can acquire 100,000 A-scans per second in both healthy and diseased eyes. The volumetric OCT scans can be acquired over a 3 × 3-mm field of view in approximately 4 s of the total OCT scan time. Each B-scan position is repeatedly scanned 4 times. The examination field can be enlarged to 6 × 6 mm (Triton SS OCT and Atlantis) or even to 12 × 9 mm (Atlantis prototype only) [36]. The algorithm used is called OCTA Ratio Analysis (OCTARA). It is based on a ratio calculation in which the full spectrum is kept intact; therefore, the axial resolution is preserved. This method provides advantages over differentiation-based approaches while possessing improved sensitivity over methods based on amplitude decorrelation [36]. Stanga et al. studied diabetic maculopathy and PDR with the Triton SS OCT platform. The authors concluded that it provides additional information regarding the localization and morphology of NV in the optic disc (NVD) and in more than half of the NV elsewhere (NVE), suggesting that it is a noninferior technique to study posterior pole alterations compared with fluorescein angiography [37].

OCT angiography can assess retinal and choroidal microcirculation, identifying subsequent anatomical findings in many retinal diseases, including DR [5, 6, 15, 25, 30, 32]. The technique allows the study of retinal deep plexi, while conventional fluorescein angiography only shows the superficial plexus. Thus, OCT angiography has been used to help diagnose DR-associated complications, but further reports are needed to expand our understanding of DR pathogenesis.

## Diabetic retinopathy R and OCT angiography

Numerous clinical findings related to DR have been identified using OCT angiography [6, 20, 38, 39]. Structural OCT angiograms identified different lesions in singular stages of DR [6]. OCT angiography is not dependent on contrast injection, which helps to provide detailed information regarding capillaries without fluorescein leakage [5, 20].

**Fig. 1:**
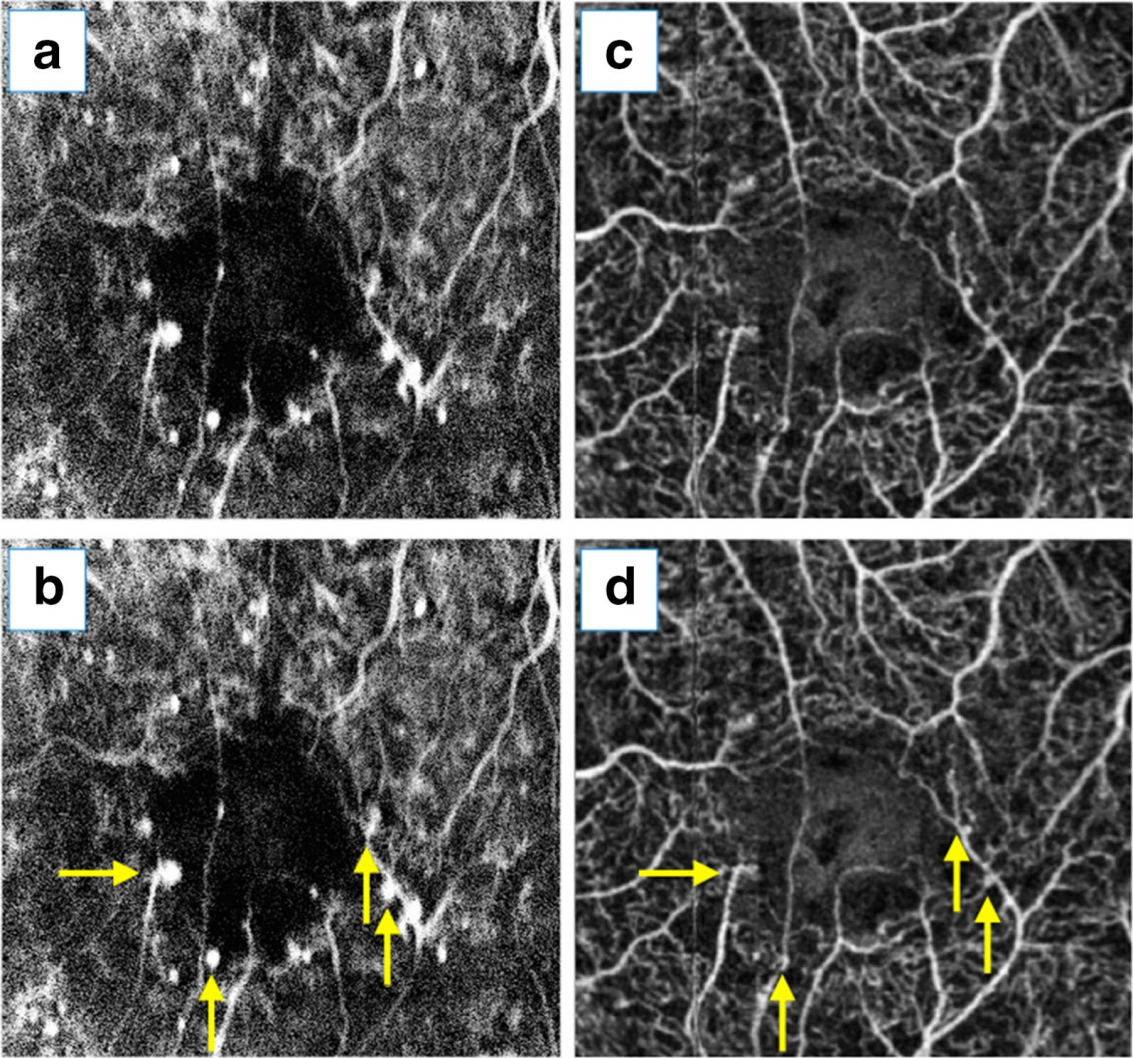
Microaneurysm assessment using OCT angiography. This image was obtained from a diabetic patient and demonstrates microaneurysms. He was subjected to fluorescein angiography (**a**, **b**) and OCT angiography (**c**, **d**). Microaneurysm topography was compared using conventional angiography (**b**) and a 3 × 3-mm OCT angiogram obtained from SCP (**d**) (*yellow arrows*)

Ishibazawa et al. [6] concluded that OCT angiographic techniques could be used to study the origin of microaneurysms, describing them as demarcated saccular or fusiform shapes of focally dilated capillary vessels in the inner retina (Fig. 1). Despite this improved identification, reports have demonstrated that the number of microaneurysms was significantly lower than that obtained by conventional angiography but also that OCT angiography had the added benefit of localizing these lesions to their exact intraretinal depths [13, 39]. Miwa et al. [12], using OCT angiography, detected only 41.0 ± 16.1% of the microaneurysms identified in the fluorescein angiography images. Using 6 × 6-mm OCT angiograms, some authors were unable to reliably identify microaneurysms, probably due to their relatively low flow and scan concentrations [5]. Another possible reason for this result may be the focal staining of the capillary walls or surrounding tissues in the microaneurysms that clearly identifies them in the fluorescein angiography images, despite OCT angiography, because these factors are independent of erythrocyte movement [12].

**Fig. 2:**
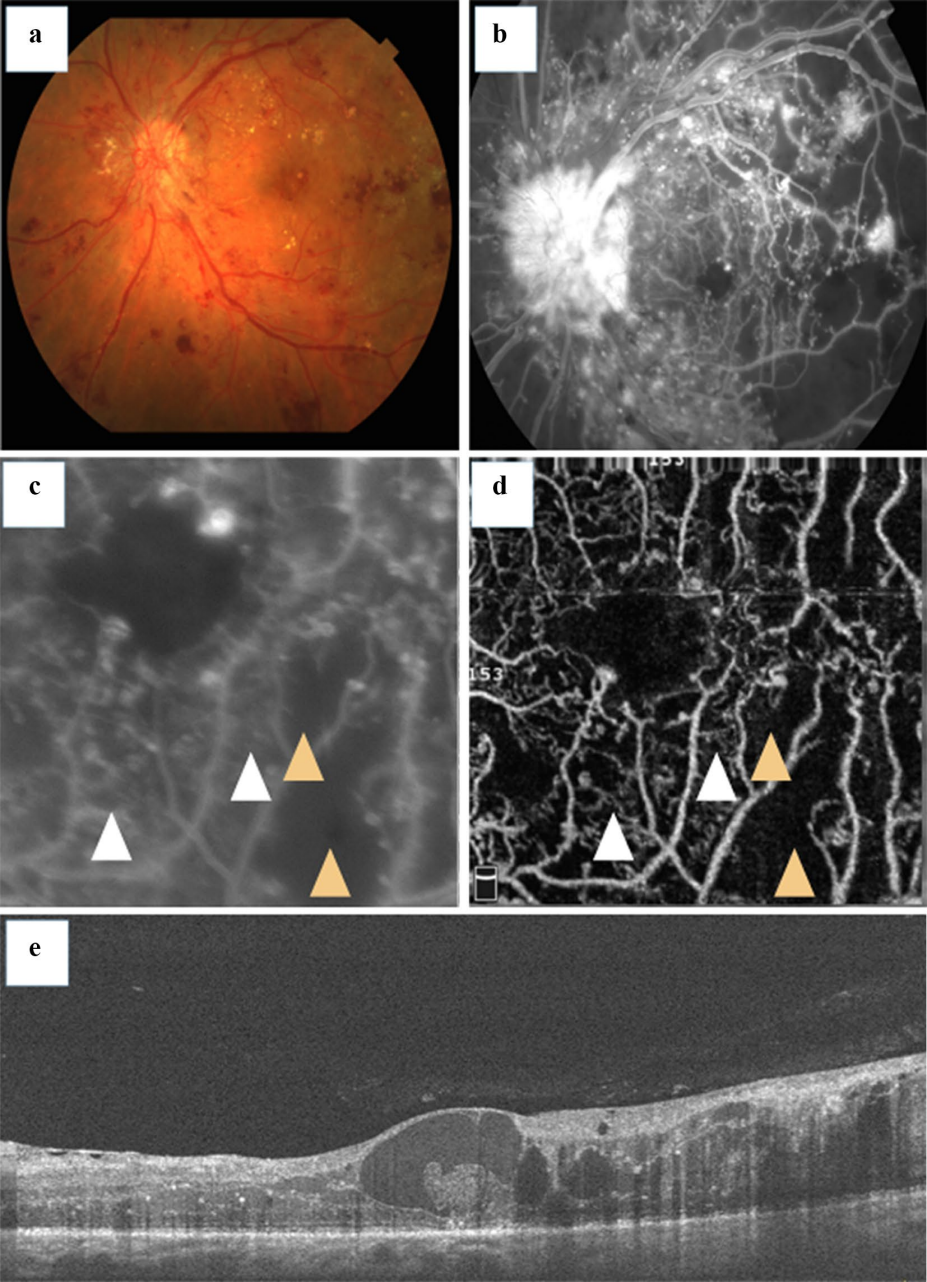
Intraretinal microcirculation and capillary nonperfusion. This patient was diagnosed with PDR. Fundus photograph and fluorescein angiography (**a**, **b**). Images of 3 × 3-mm fluorescein angiography (**c**) and OCT angiography were obtained (**d**). The *white arrowheads* indicate superficial collaterals with adjacent nonperfusion areas (*orange arrowheads*) (**c**). The corresponding SD OCT B-scan is also shown (**e**)

Other DR clinical findings, such as arteriolar wall staining, neovascularization, and intraretinal microvascular abnormalities (IRMA), have divergent appearances on OCT angiography and fluorescein angiography. Wall staining and arteriolar narrowing have been illustrated as intense attenuation of microcirculation caliber on OCT angiography. IRMA, depicted as dilated terminal vessels surrounded by capillary loss, were similarly identified with OCT angiography and fluorescein angiography (Fig. 2) [5]. OCT angiograms clearly visualized new vessels on the disc (NVD) that persisted as spiral, looped, and irregular structures after initial anti-VEGF therapy [6]. Retinal NV is mostly detected by observing the flow signal above the internal limiting membrane. Hyperfluorescent lesions on fluorescein angiography that appeared indistinguishable from an MA were identified as NV using OCT angiography. This information may help us to understand why some patients with PDR and vitreous hemorrhage do not have a definitive NV on fluorescein angiography, as long as this method does not always identify all NV [5].

## Diabetic macular ischemia on OCT angiography

Diabetic macular ischemia (DMI) is associated with functional retinal damage, and its diagnosis predicts DR progression [4, 19, 40, 41]. It is characterized by the enlargement and disruption of the foveal avascular zone (FAZ) and by retinal capillary loss in other noncontiguous areas of the macula (capillary dropout) (Fig. 2) [41]. It has been postulated that the selective loss of pericytes and thickening of the basement membrane in retinal capillaries occur due to the effects of chronic hyperglycemia, leading to capillary occlusion, one of the characteristics findings of DMI [14]. Sim et al. [15, 40] demonstrated that approximately 41% of patients with DR in a tertiary hospital setting had some degree of macular ischemia. DMI is usually associated with reduced visual acuity (VA) in the eyes with moderate to severe grades of ischemia, but VA is preserved in milder grades of ischemia [10, 41, 42].

Because antiangiogenic therapy has become one of the treatments of choice for managing DR, the diagnosis of DMI has become more important, especially because antiangiogenic therapy can obscure the clinical findings related to ischemia progression [4, 19, 40, 41, 43]. Angiogenic factors, mainly VEGF, are believed to play a protective role in ischemic retinal disorders, improving volumetric blood flow and protecting retinal cells against apoptotic conditions [43]. Nevertheless, Campochiaro et al. [44] concluded that monthly injections of ranibizumab (Lucentis; Genentech, San Francisco, US) could diminish, but not completely prevent, retinal capillary closure in patients with DME.

**Fig. 3:**
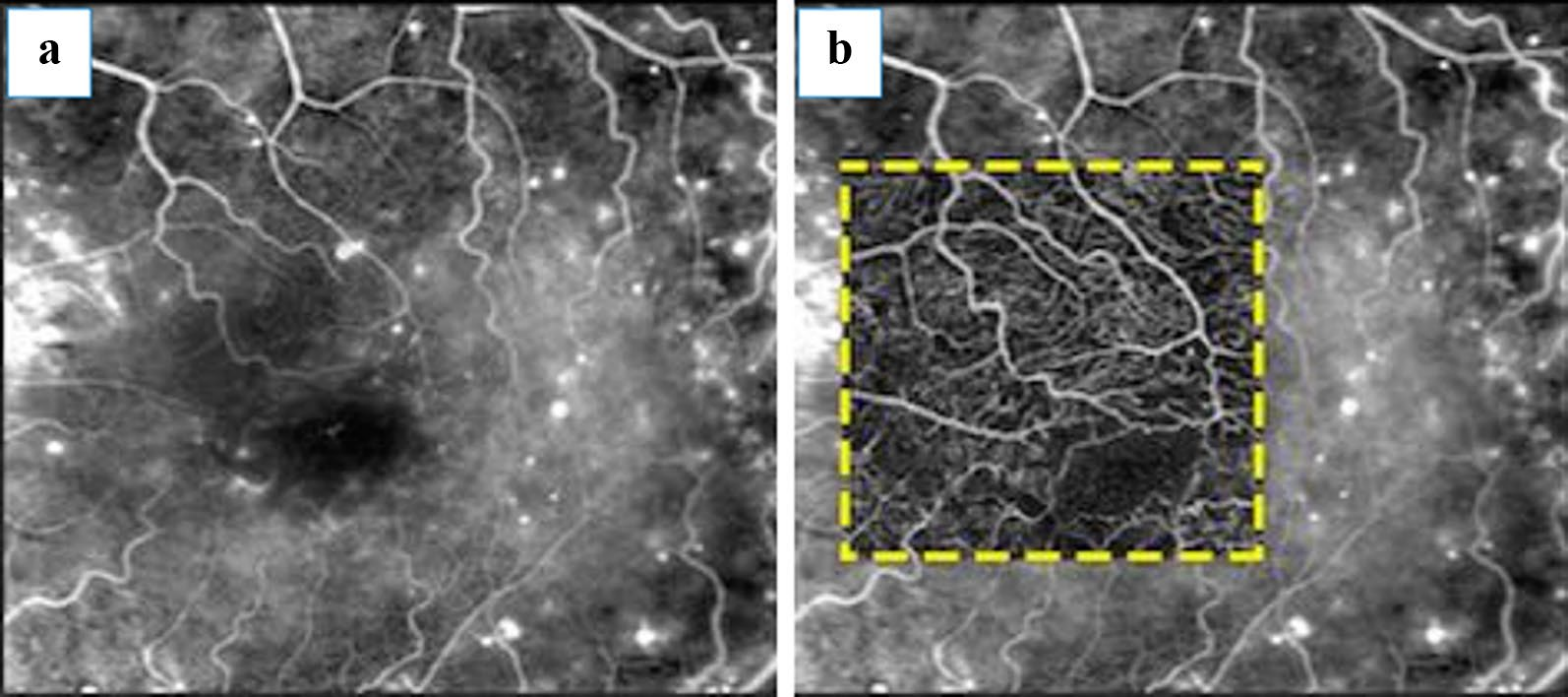
Improving retinal imaging in DR on OCT angiography. This fluorescein angiography image (**a**) is overlapped by a 3 × 3-mm OCT angiogram obtained from the SCP (**b**), displaying better details in the absence of dye staining and leakage

Fluorescein angiography was introduced in 1961, and it has become the gold standard imaging modality for assessing the macular capillary network and its pathological conditions, such as macular ischemia [30, 43]. However, this method cannot distinguish between superficial and deep capillary plexi, as it displays in two-dimensional images the capillary bed of the superficial vascular plexus (Fig. 3) [20, 22, 30, 45].

Recent publications have shown that OCT angiography clearly depicts nonperfusion in DR [5, 6, 12, 20]. It provides an objective automated study of macular capillary nonperfusion as a potential sign of central ischemia [4]. Using OCT angiography with SSADA in healthy participants (n = 105), Wang et al. measured a mean FAZ of 0.35 ± 0.12 mm^2^. In addition to DMI, a larger FAZ was found in subjects with shorter axial lengths, hyperopia, increased subfoveal choroidal thicknesses, thicker lenses, shallower anterior chamber depths, and other characteristics [45]. Bradley et al. [15] demonstrated moderate agreement between the DMI grading results for OCT angiography (AngioVue platform) and fluorescein angiography in 24 diabetic patients using standard ETDRS protocols.

**Fig. 4:**
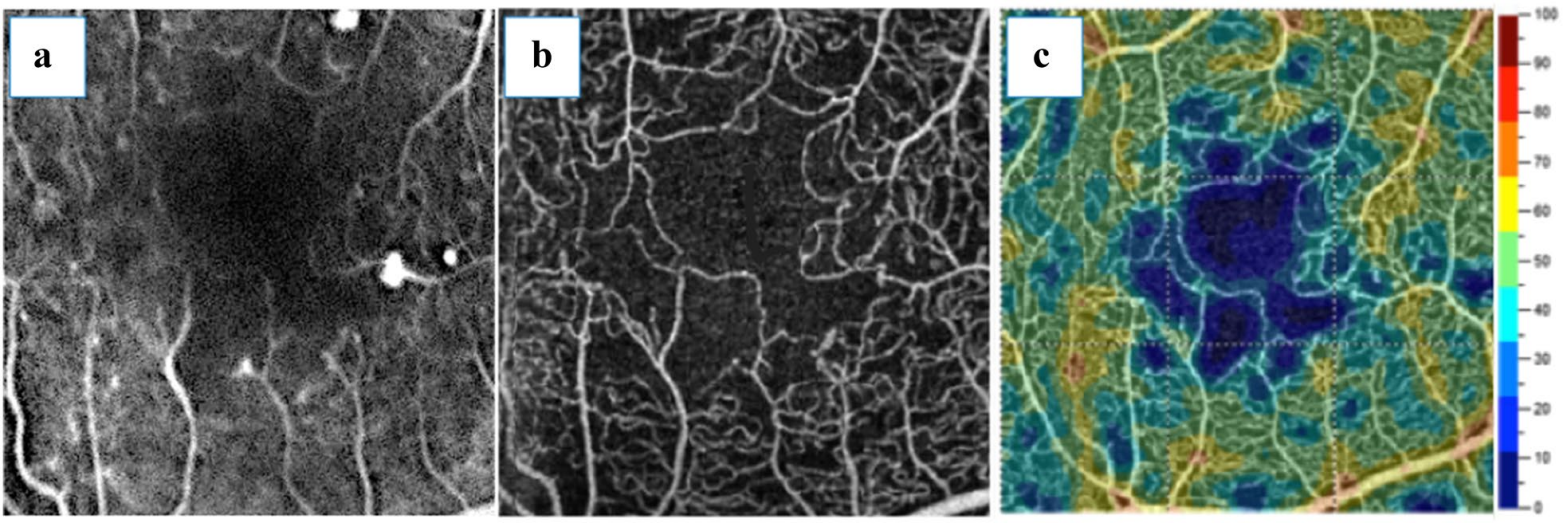
Perifoveal perfusion density. Diabetic macular ischemia is demonstrated on fluorescein angiography (**a**) and a 3 × 3-mm OCT angiogram obtained from the SCP (**b**). Corresponding perfusion density mapping is represented by *color*-*coded* topographic maps with quantitative data (**c**)

Hwang et al. [5] studied patients with proliferative DR (PDR), identifying FAZ enlargement and irregularities, as well as areas of capillary dropout. Miwa et al. [12] demonstrated that FAZ areas in OCT angiograms in the superficial layer were smaller than those in the fluorescein angiography images and were correlated with each other, agreeing with the finding that OCT angiography images often delineate the vascular structures in the nonperfused areas shown by fluorescein angiography. Usually, retinal microcirculation imaging is improved on OCT angiography despite fluorescein angiography (Fig. 4).

**Fig. 5:**
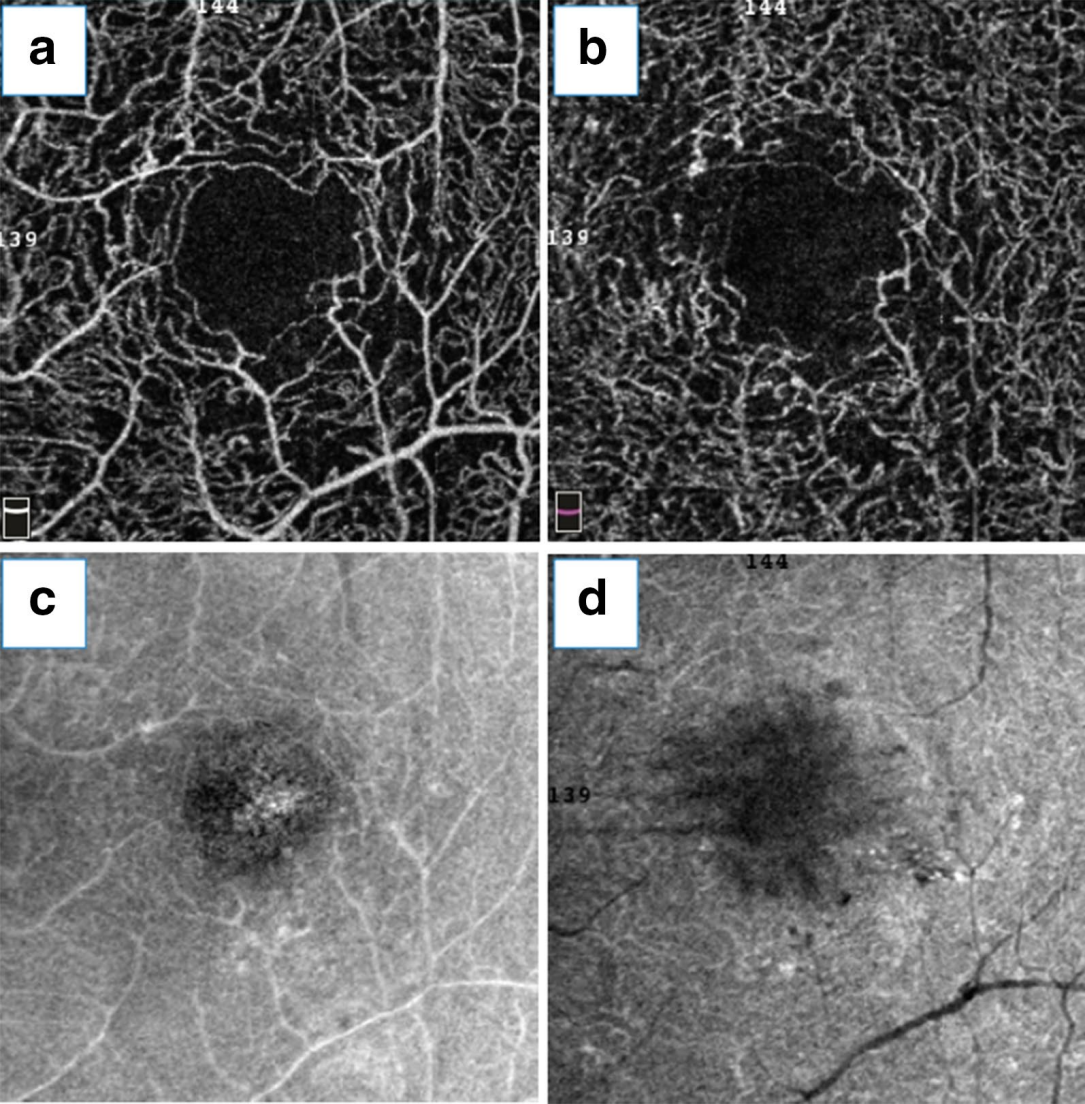
Diabetic macular ischemia and OCT angiography. This patient was diagnosed with diabetic macular ischemia, represented by superficial capillary plexus (SCP) (**a**) and deep capillary plexus (DCP) (**b**) OCT angiography images. *En face* OCT angiograms from the SCP and DCP (**c**, **d**, respectively) are also displayed

The identification of DCP by OCT angiography plays an important role in the setting of DMI (Fig. 5). Experimental studies have shown that the DCP contributes to photoreceptor inner segment oxygen requirements (10–15%), particularly during dark adaptation [46]. Recently, some authors have postulated that patients with DCP nonperfusion had an outer retina disruption on SD OCT in DMI, corresponding to areas of capillary nonperfusion at the level of the DCP [47, 48]. Yi et al. [49] demonstrated that during systemic hypoxia, the inner retina contribution to the metabolic needs of the outer retina microcirculation become even more significant because the choroidal vasculature fails to autoregulate its blood supply in the hypoxic setting. Couturier et al. [13] concluded that SCP capillary rarefaction was better identified on OCT angiography. Nevertheless, the DCP nonperfused areas were observed in only 35% of the subjects studied.

**Fig. 6:**
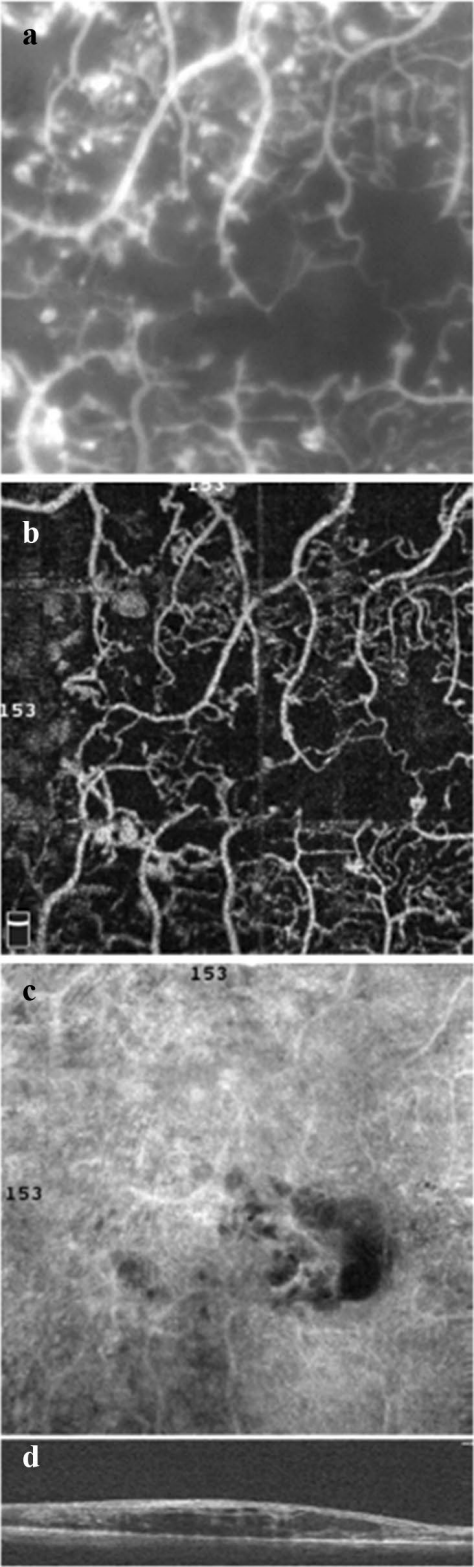
Ischemic DME. Fluorescein angiography (**a**) compared with the 3 × 3-mm SCP OCT angiogram (**b**). DCP (**c**) and corresponding SD OCT B-scan (**d**)

Recently, a novel quantitative graphic mapping technique for studying and displaying retinal vascular perfusion density using OCT angiography was developed. The authors applied software to images obtained from the Optovue SSADA algorithm that translates the OCT angiogram for each layer into qualitative and quantitative data using perfusion density analysis. Diabetic patients had significantly poorer capillary perfusion on SCP, DCP, and choriocapillaris than the controls in both the 3 × 3-mm and 6 × 6-mm settings. Subgroup analysis showed that normal subjects had higher capillary perfusion rates than patients diagnosed with mild non-proliferative DR (NPDR) (Fig. 6) [8].

## General perspectives and critical improvements

Previously, the available OCT angiography devices were used to provide high-resolution OCT angiograms in patients with retinal diseases, including diabetic patients. Most of them apply the motion contrast concept to generate volumetric blood flow information, providing angiographic images after comparing sequential B-scans. However, other techniques have been described to image retinal blood flow, such as SS OCT, PV OCT, and Doppler OCT.

Doppler OCT has been used in conjunction with available SD OCT instrumentation. The Doppler effect is based on signals back-scattered from moving particles [24, 26, 50–52]. Doppler OCT is particularly useful for comparing depth scans, using this information to calculate the flow component parallel to the imaging direction (called axial flow) [51, 53]. Nevertheless, without dedicated scanning protocols, this technique is limited to the detection of strictly slow flow [54]. Furthermore, the inherent disadvantage of the angle dependence of the Doppler phenomenon has prevented it from becoming a reliable clinical tool for imaging retinal blood flow [52].

PV OCT has been used to image retinal circulation by Fingler et al. and Kim et al. The method differs from Doppler OCT by generating increased SD OCT imaging speeds, maintaining the previously demonstrated ability to display fast and slow blood flow independent of imaging orientation [51].

The SS OCT technique utilizes a 1050-nm wavelength instead of the 800-nm wavelength used in most commercially available SD OCT instruments [22, 33, 39, 52]. There has been active research interest in exploring SS OCT systems at the 1-µm wavelength range for imaging ocular posterior segments, offering the advantages of improved sensitivity roll-off and increased penetration depth into the choroidal tissue, higher resolution images, and a faster acquisition time than conventional SD OCT devices [52]. Thus, SS OCT angiography is a promising method that has numerous algorithms [33, 52]. The Massachusetts Institute of Technology has developed an OCT angiography prototype using this technology with a faster acquisition rate than that of commercially available SS OCT angiography instruments. This ultra-high-speed prototype employs a vertical cavity surface emitting laser (VCSEL) operating at a 1060-nm wavelength that allows increased light penetration into pigmented tissues and improved choroidal blood flow visualization compared with the light source used in SD OCT. This SS OCT angiography system obtains 500 × 500 A-scans at 400,000 A-scans per second in approximately 3.8 s [22].

Some commercial OCT angiography manufacturers are starting to include automated algorithms for mapping capillary density in their instrument software [8, 22]. Because OCT angiography imaging is based on motion contrast, it is properly suited for assessing macula perfusion status, such as identifying regions with no detectable flow [8]. The follow-up image capture and intersession repeatability of automated vessel density measurements have been objectively demonstrated in healthy individuals. Further reports are needed in diabetic patients [55, 56]. In addition to mapping capillary perfusion, novel methods that allow the assessment of relative blood flow speeds have been developed. Variable interscan time analysis (VISTA), an OCT angiography imaging and analysis technique, was developed to overcome the limitation that typical OCT angiography does not directly measure blood flow speeds. Ploner et al. [57] concluded that abnormal flow speed was detected in several macular disorders, including DR.

OCT angiography must be properly compared with fluorescein angiography, as long as both methods have some limitations. OCT angiography requires that the patient fixate precisely for several seconds, whereas a useful fluorescein angiographic frame can be obtained in a fraction of a second [30]. In its current state, most commercially available OCT angiography systems suffer from motion artifacts and a relatively small field of view, which can be improved with further development efforts [4–6, 20, 25]. This instrument uses image alignment based on scanning laser ophthalmoscope (SLO) images obtained immediately prior to image capture. This eliminates the relatively long image acquisition time necessary for image capture when the automatic tracking function is used [56].

## Conclusions

OCT angiography is a relatively new and promising imaging technique that uses SD OCT or SS OCT for the 3D visualization of the retinal and choroidal microcirculation without the need for dye injection. While fluorescein angiography is still considered the gold standard for imaging retinal vasculature in vivo, OCT angiography is a non-invasive, relatively fast imaging study that can be performed alongside routine OCT imaging. Several studies have demonstrated its applicability in diabetic patients, such as providing a prognosis and assessing treatment effects. Presently, OCT angiography allows the study of the retinal vascular bed and discriminates between superficial and deep retinal vascular plexi, which cannot be portrayed by conventional fluorescein angiography. In contrast, fluorescein angiography techniques are still superior in identifying slow blood flow structures such as microaneurysms. In summary, OCT angiography may provide images with greater detail regarding macular status and may become a novel imaging technique for the diagnosis of DMI in conjunction with fluorescein angiography in the management of DR.
